# The Evolution of Collagen Fiber Orientation in Engineered Cardiovascular Tissues Visualized by Diffusion Tensor Imaging

**DOI:** 10.1371/journal.pone.0127847

**Published:** 2015-05-27

**Authors:** Samaneh Ghazanfari, Anita Driessen-Mol, Gustav J. Strijkers, Frank P. T. Baaijens, Carlijn V. C. Bouten

**Affiliations:** 1 Soft Tissue Biomechanics and Engineering, Department of Biomedical Engineering, Eindhoven University of Technology, Eindhoven, the Netherlands; 2 Biomedical NMR, Biomedical Engineering and Physics, Academic Medical Center, Amsterdam, the Netherlands; University Hospital of Modena and Reggio Emilia, ITALY

## Abstract

The collagen architecture is the major determinant of the function and mechanical behavior of cardiovascular tissues. In order to engineer a functional and load-bearing cardiovascular tissue with a structure that mimics the native tissue to meet *in vivo* mechanical demands, a complete understanding of the collagen orientation mechanism is required. Several methods have been used to visualize collagen architecture in tissue-engineered (TE) constructs, but they either have a limited imaging depth or have a complicated set up. In this study, Diffusion Tensor Imaging (DTI) is explored as a fast and reliable method to visualize collagen arrangement, and Confocal Laser Scanning Microscopy (CLSM) was used as a validation technique. Uniaxially constrained TE strips were cultured for 2 days, 10 days, 3 and 6 weeks to investigate the evolution of the collagen orientation with time. Moreover, a comparison of the collagen orientation in high and low aspect ratio (length/width) TE constructs was made with both methods. Both methods showed similar fiber orientation in TE constructs. Collagen fibers in the high aspect ratio samples were mostly aligned in the constrained direction, while the collagen fibers in low aspect ratio strips were mainly oriented in the oblique direction. The orientation changed to the oblique direction by extending culture time and could also be visualized. DTI captured the collagen orientation differences between low and high aspect ratio samples and with time. Therefore, it can be used as a fast, non-destructive and reliable tool to study the evolution of the collagen orientation in TE constructs.

## Introduction

Collagen is a major fibrous protein of the extracellular matrix (ECM). It is the main load-bearing constituent of cardiovascular tissues and provides the tissue with the capacity to withstand hemodynamic forces [1]. Different collagen architectures, including collagen organization, cross-linking and collagen content, result in variation in biomechanical behavior between and within tissues [2–4].

Tissue engineering of load-bearing cardiovascular tissues requires the complete understanding of the mechanisms that underlie the formation and evolution of the fibrous architecture of the ECM to be able to replicate the structure and functional behavior of native tissues [5]. Tissue cells generate traction forces when tissue is developing and the exerted force on the ECM is demonstrated more predominant in the direction of cell alignment [6–8]. The resulting mechanical stress applied to the tissue and consequent tissue compaction causes reorganization of collagen fibers, especially when the tissue is constrained, and consequently influences the mechanical properties of the tissue [9–11]. The traction forces generated by the cells may cause the collagen to align in the direction of the cells [12–16].

A method is required to visualize and understand the collagen organization within the whole tissue to gain a complete understanding of the fibrous organization and to explain the functional and mechanical behavior of the tissue and to develop realistic mathematical and numerical models. Several methods are used for collagen visualization, such as microscopic elliptical polarimetry [17], small light scattering [18], polarized light microscopy [19], and X-Ray diffraction [20]. The imaging depth of these methods is rather limited and they merely provide global information about the collagen orientation. The resolution of second harmonic generation (SHG) microscopy is higher compared to the previously mentioned methods, but the technique is not suitable to visualize the immature newly formed collagen fibers in TE constructs. To image these newly formed fibers, a collagen binding fluorescent probe (CNA35) was developed by Nash-Krahn et al. [21]. The use of this probe in combination with CLSM allows to obtain 3D high resolution microscopy images when the tissue is not thicker than a few hundred micrometers. If the TE construct is thicker, it has to be sliced and the registration of sequential images will be needed in order to make the 3D structure [22]. This is a very time-consuming and destructive procedure and the tissue structure might be influenced by the freezing and slicing procedures [4, 23]. DTI is suggested here as a fast and non-destructive method to visualize the collagen structure in engineered tissues. The main disadvantage of the DTI method compared to CLSM is its limited spatial resolution. Thus, ‎DTI analysis becomes unreliable in regions where fiber orientations vary strongly at a sub-voxel ‎level. ‎

Diffusion tensor imaging (DTI) is an imaging modality that provides information on the directional anisotropy of water diffusion [24]. Water diffusion is faster along the fibers than across them. Based on the diffusion directions, the diffusion tensor, which contains the information about the 3D fiber orientation, is generated. A diffusion ellipsoid is used to show the geometric representation of the diffusion tensor. Ellipsoid size, shape and orientation are associated with the mean diffusivity and the diffusion anisotropy, respectively [25]. Fiber tractography is used to visualize the fiber structure from the 3D diffusion tensor. In each voxel, the fiber tract direction is parallel to the eigenvector associated with the largest eigenvalue [26]. This method has been used to evaluate the fibrous structure of several native tissues and here we will explore the feasibility of this method in TE constructs [26–31]. Recently, we have demonstrated the use of DTI to evaluate the collagen orientation in the carotid artery [32].

The aim of this study was to evaluate collagen orientation in TE constructs, fabricated from human vascular derived cells seeded into a rapid degrading synthetic scaffold, over time (2 days, 10 days, 3 and 6 weeks) and with different aspect ratios (1:3 and 1:5). DTI was performed on the TE constructs and confocal laser scanning microscopy (CLSM) in combination with the CNA35 probe was used as a validation method. To understand the collagen orientation, tissue compaction was evaluated by histology.

## Materials and Methods

### Cell and tissue culture

**Fig 1:**
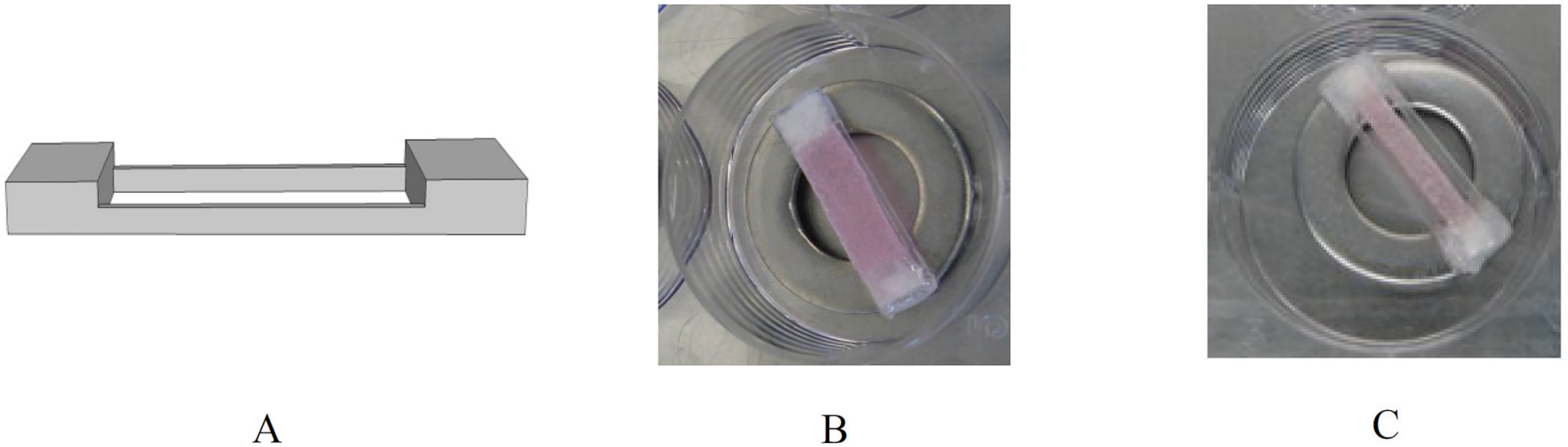
(A) Schematic drawing of the polycarbonate frame, (B) low (size: 25×5×1 mm) and (C) high (size: 25×3×1 mm) aspect ratio TE strips glued to the frame just after seeding.

Human vascular-derived cells were harvested from the *vena saphena magna* as described earlier [33]. The vascular tissue, which was considered surgical waste material, was kindly supplied by the Catharina Hospital Eindhoven following a no-objection procedure: patients were informed about the potential use of rest material for scientific research purposes. Material was handed over without any patient-specific information except for gender according to the procedure for secondary use of ‎patient material as described in the Dutch code of conduct for responsible use of ‎patient material. According to the Dutch medical scientific research with human subjects act ‎‎(WMO), secondary use of patient material does not need review by a Medical Ethics ‎Examination Committee. ‎Cells were maintained at 37°C in advanced Dulbecco’s Modified Eagle Medium (DMEM; Invitrogen, Breda, The Netherlands), supplemented with 10% Fetal Bovine Serum (FBS; Greiner Bio one, Frickenhausen, The Netherlands), 1% GlutaMax (Gibco), and 1% penicillin/streptomycin (Lonza, Basel, Switzerland). Rectangular scaffolds with high aspect ratio (strips size: 25×3×1 mm; seeding area: 15×3×1) and low aspect ratio (strips size: 25×5×1 mm; seeding area: 15×5×1) were cut from a rapid degrading non-woven polyglycolic acid mesh (PGA; specific gravity, 70 mg/cm^3^; Cellon, Bereldange, Luxembourg) and were coated with poly-4-hydroxybutyrate (P4HB; obtained as a part of collaboration with University Hospital Zurich) to maintain mesh integrity. Tiny frames were made of polycarbonate to enable placement of the constrained samples in the MRI scanner (Fig 1A). Rectangular scaffolds were constrained in the longitudinal direction by gluing them to the frames with polyurethane-tetrahydrofuran (15% wt/vol) (Fig 1B and 1C). The frames were attached to metal rings to submerge the constructs within the medium. The scaffolds were incubated in 70% ethanol and washed in PBS afterwards to achieve sterilization. They were incubated in tissue engineering medium (cell culture medium supplemented with L-ascorbic acid 2-phosphate (0.25 mg/ml, Sigma, USA)) 24 hours before seeding to facilitate cell attachment. The strips were seeded with passage 7 human vascular-derived cells with a density of 15 million cells per cm^3^ using fibrin gel as a cell carrier [34]. The strips with high aspect ratio were cultured for 10 days, 3 and 6 weeks to evaluate the effect of culture time on the collagen orientation. This was done for both DTI and CLSM imaging methods. To evaluate the tissue compaction by histology, 3 strips were cultured for 2 days to compare their geometry, representing the original shape at that stage, with other histology groups cultured for 10 days, 3 and 6 weeks. The TE constructs with lower aspect ratio were cultured for 3 weeks to investigate the influence of aspect ratio on the collagen orientation. Both configurations were cultured under similar conditions. CLSM, DTI and histology were performed on 3 independent samples per configuration per time point. Tissue engineering medium was replaced twice a week.

### CLSM and image analysis

For CLSM, constrained TE strips were incubated with 3μM CNA35-OG488 probe overnight to stain the collagen fibers. They were washed with phosphate-buffered saline (PBS) before imaging. Images were recorded with a Zeiss LSM 510 Meta laser scanning microscope (Carl Zeiss, Germany) coupled to an inverted Axiovert 200 microscope (Carl Zeiss, Germany). Fluorescent signals of CNA35-OG488 are detectable by the microscope and show broad spectra at 520 nm. Tile scans (10×25 tiles) were carried out across the strips area to obtain the collagen orientation of the whole strip. Microscope stage positioning was performed automatically by programming the computerized x—y stage. The increment of the z-axis optical section was 3 μm. The mean scan time for one side of each strip was about 9 hours. Due to the imaging depth limitation of CLSM, the other side of the ‎samples was visualized at randomly selected regions to check the similarity of the collagen orientation through the whole strip.

To reconstruct collagen architecture in the strips, the tiles were stitched together in Matlab (Mathwork, USA). As fully explained by Daniels et al., the collagen orientation analysis of microscopy images was obtained by an algorithm developed in Mathematica (Wolfram, USA) [35]. The analysis was performed on each tile separately. The local collagen fiber orientation was calculated based on the principal curvature directions, which were determined from the Hessian’s matrix and were calculated per pixel. A histogram was obtained from the program showing all local fiber orientations in each tile. Moreover, the mean angle (α) and dispersity (r) of the fibers distribution were calculated for each image based on circular statistics [36–38]. The mean angle was calculated with respect to the perpendicular axis.‎ The mean vector was obtained by averaging the decomposed sine and cosine vector components of all unit vectors of the histogram. The length of the mean vector represents the fiber dispersity (r). If r is zero, fibers are oriented randomly and if r is 1, fibers are fully aligned. The mean angle and dispersity of the fiber orientations in each tile is represented in the form of ellipses. To evaluate and examplify this method randomized and aligned fibers were depicted and analyzed using the quantification algorithm. For quantitative comparison of CLSM with DTI data, we analyzed an equivalent rectangular region of 2700 × 900 pixels, containing 12 tiles, from the central region (x-y plane) of high and low aspect ratio constructs cultured for 3 weeks and from the compacted area of high aspect ratio constructs cultured for 6 weeks.

### DTI and collagen orientation quantification

**Fig 2:**
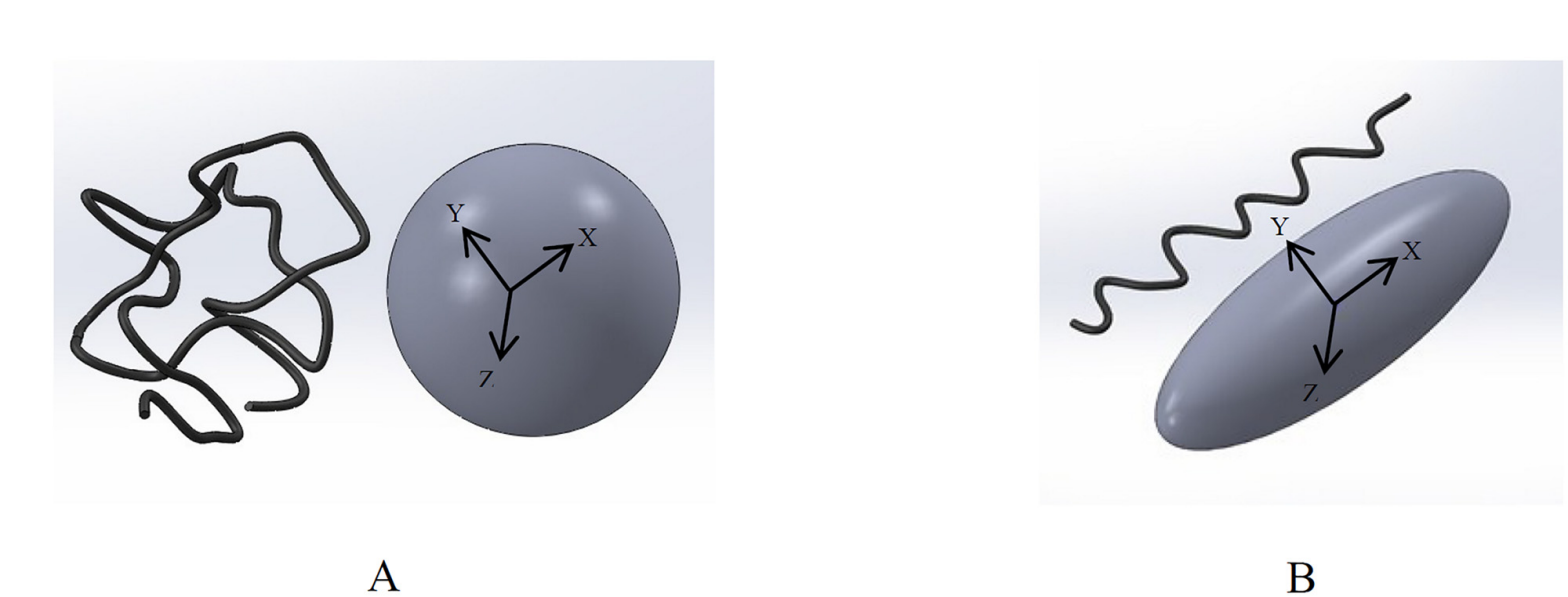
Geometric representation of the diffusion tensors, showing isotropic (A) and anisotropic (B) diffusion.

The method for DTI scanning and collagen fiber analysis was described before [32]. In brief, constrained TE strips were placed in a cryovial (Sigma, USA) and embedded in 4% type VII agarose (Sigma, USA). The scanning was performed with a 6.3 T horizontal-bore MRI scanner (Bruker, Ettlingen, Germany) equipped with a shielded gradient set with a maximum gradient of 450 mT/m on all 3 axes. MRI signal transmission and reception was done with a home-built send-and-receive solenoid radiofrequency (RF) coil with a diameter of 10 mm and length of 15 mm. Diffusion-tensor-imaging was performed using a 3D spin-echo sequence with 2 pulsed field gradients for diffusion weighting placed symmetrically around the 180° pulse. Sequence parameters were: echo time = 20 ms, repetition time = 1000 ms, number of signal averages = 1, field-of-view = 25.6×25.6×25.6 mm^3^, acquisition matrix = 128×64×64, reconstruction matrix = 128×128×128, spatial resolution = 200×200×200 μm^3^, 10 diffusion directions, and b-value = 900 mm^2^/s. Diffusion directions and b-value were optimized according to the procedures outlined by Jones et al. [39]. The acquisition time for each strip was 11 hours 25 minutes. Fiber tractography was performed by DTI software developed in-house [40]. ‎Using this software, 3D ellipsoids were used as the geometric representation of the diffusion tensors. The shape of the ‎ellipsoids is defined by the eigenvalues and eigenvectors of the diffusion tensors (Fig 2). If the ‎diffusion tensor is isotropic, the diffusion ellipsoid is a sphere (Fig 2A), and if the diffusion ‎tensor is anisotropic, it becomes an elongated ellipsoid (Fig 2B), where the long-axis of the ‎ellipsoid aligns with the principal fiber orientation.‎ The tensor orientation was represented using a red-green-blue (RGB) color map to indicate the major eigenvector directions. The fiber seeding region of interest was chosen based on the T2-weighted images to cover the whole strip. To compare the DTI and CLSM data, the fibers obtained by DTI were projected on the x-y plane. A rectangular region of the x-z plane, whose size and location was the same as that of CLSM, was analyzed.

### Histology

To assess tissue compaction, TE strips were evaluated by histology. Constrained TE samples were fixed in 3.7% formaldehyde in PBS. Subsequently, the strips were detached from the frame and embedded in paraffin transversely. Tissues were sectioned at 10 μm and stained with Haematoxylin and Eosin (H&E, Sigma, USA) to visualize the general tissue morphology. The staining was visualized under a Zeiss light microscope (Carl Zeiss). All strips were compared with the ones cultured for 2 days to define the degree of compaction.

### Statistics

Mean angle and dispersity were averaged per construct and then per group. To test statistical differences between samples with different aspect ratios and in time, two-way ANOVA using GraphPad prism 5.0 was performed (Graphpad software, CA, USA). Differences were considered statistically significant at p<0.05.

## Results

### CLSM and collagen orientation analysis

**Fig 3:**
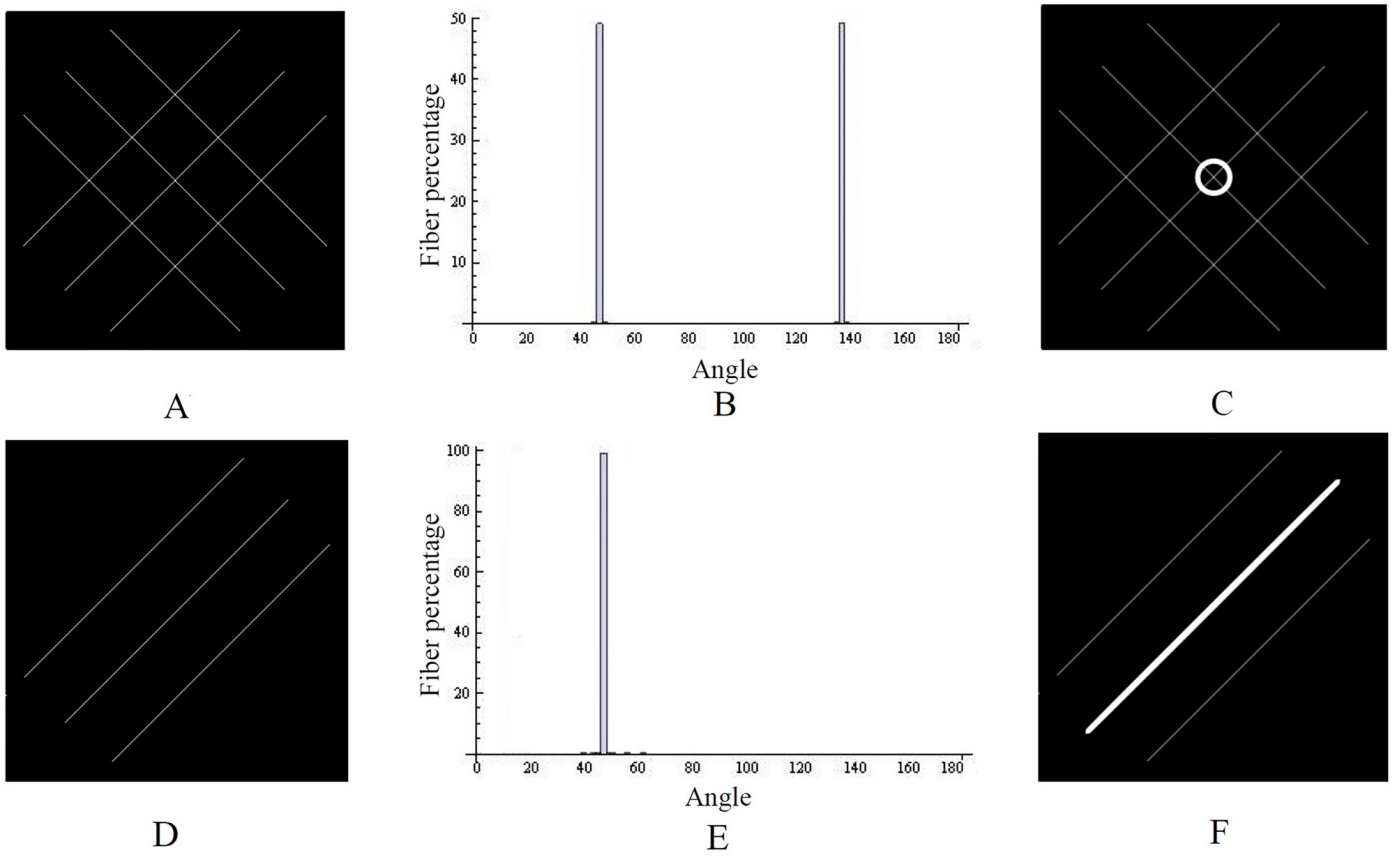
Artificial fibers with isotropic (A) and anisotropic (D) alignment, their corresponding histograms (B, E), and dispersity as ellipses (C, F). Isotropic fibers are shown as a circle and anisotropic fibers as a line.

Representative isotropic and anisotropic artificial fiber alignment images (Fig 3A and 3D), their corresponding histograms (Fig 3B and 3E), the ellipses representing the dispersity of the fiber orientations and the mean angle (Fig 3C and 3F) are shown in Fig 3. The circle in Fig 3C shows that the fibers were isotropically oriented and the line in Fig 3F shows that fiber alignment was completely anisotropic.

**Fig 4:**
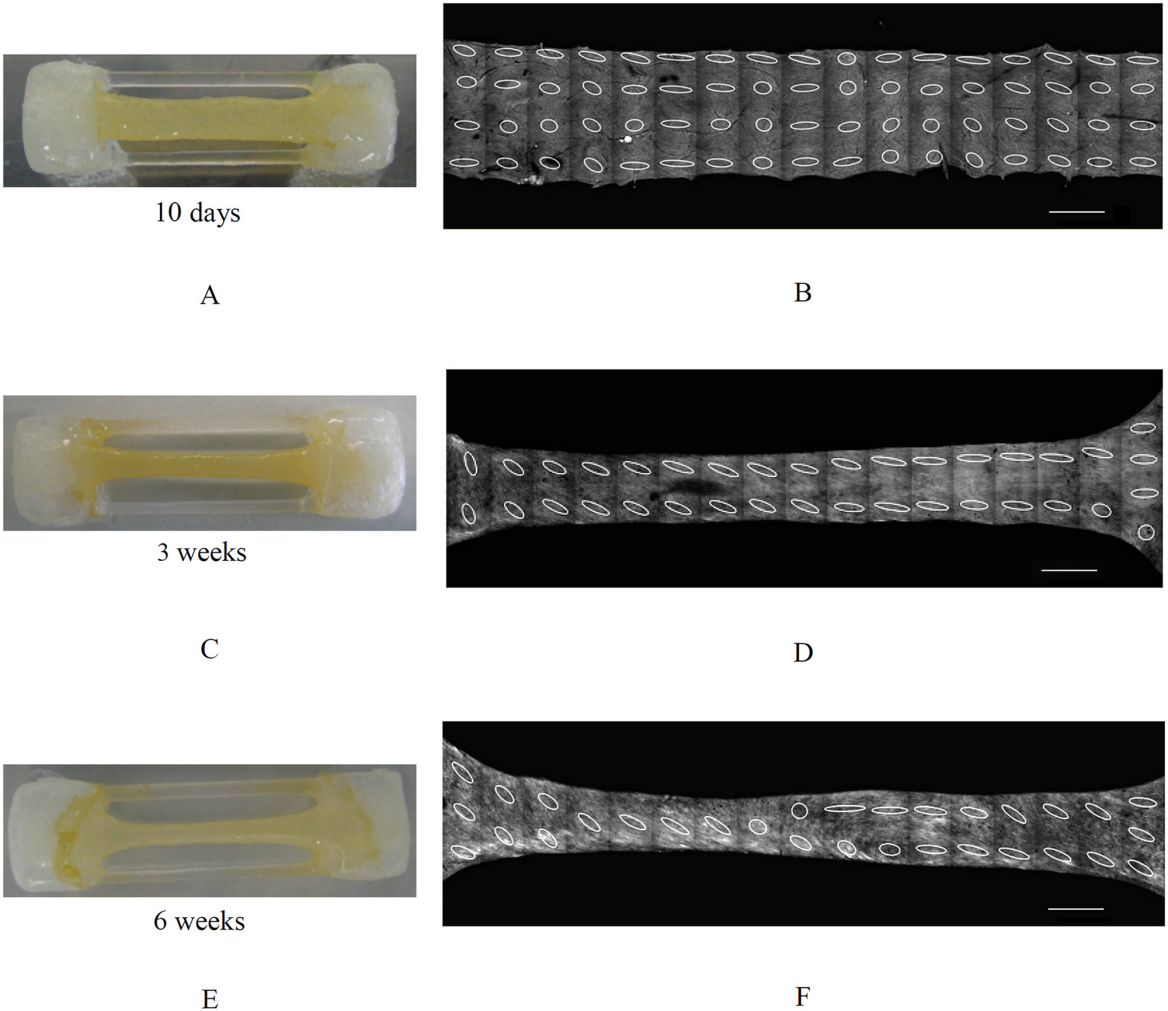
Macroscopic images of high aspect ratio TE strips cultured for 10 days (A), 3 weeks (C) and 6 weeks (E) and their corresponding CLSM images (B, D, F). (Strip size: 25×3×1 mm) collagen fiber alignment changed to the constrained direction with time and changed to the oblique direction in regions where compaction is more pronounced. Scale bars represent 1 mm.

**Fig 5:**
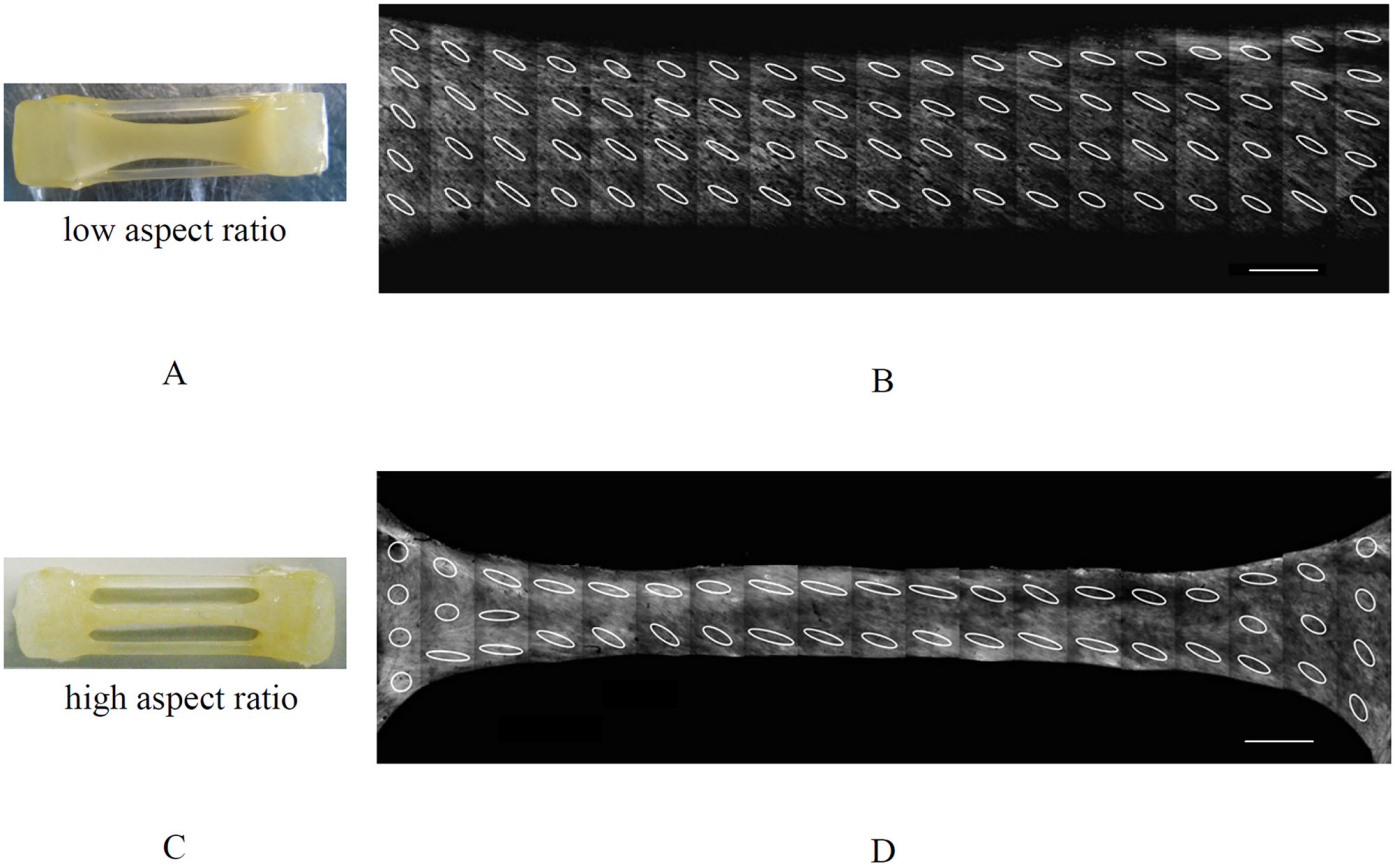
Macroscopic images of low (A) and high (C) ratio rectangular TE strips cultured for 3 weeks and corresponding CLSM images of collagen fibers (B, D). The scale of alignment is shown by ellipses for each tile. Collagen fibers aligned in the oblique direction in low aspect ratio strips after 3 weeks, while they were aligned in the constrained direction in high aspect ratio strips. Scale bars represent 1 mm.

Fig 4 shows the macroscopic pictures (Fig 4A, 4C and 4E) and their corresponding three-dimensional CLSM projections of high aspect ratio TE strips after 10 days, 3 and 6 weeks of culture. Not all collagen fibers were aligned after 10 days as is shown in Fig 4B. The fibers were aligned in the constrained direction after 3 weeks (Fig 4D) and by extending the culture time, the fiber orientation changed to oblique in the areas where the compaction in width was accentuated (Fig 4F). Macroscopic pictures and corresponding three-dimensional CLSM projections of low and high aspect ratio TE constructs after 3 weeks of culture are shown in Fig 5. Collagen fibers in low aspect ratio strips were mainly oriented in the oblique direction. In high aspect ratio strips, the fibers were mainly aligned in the constrained direction of the strip.

### DTI and fiber orientation analysis

**Fig 6:**
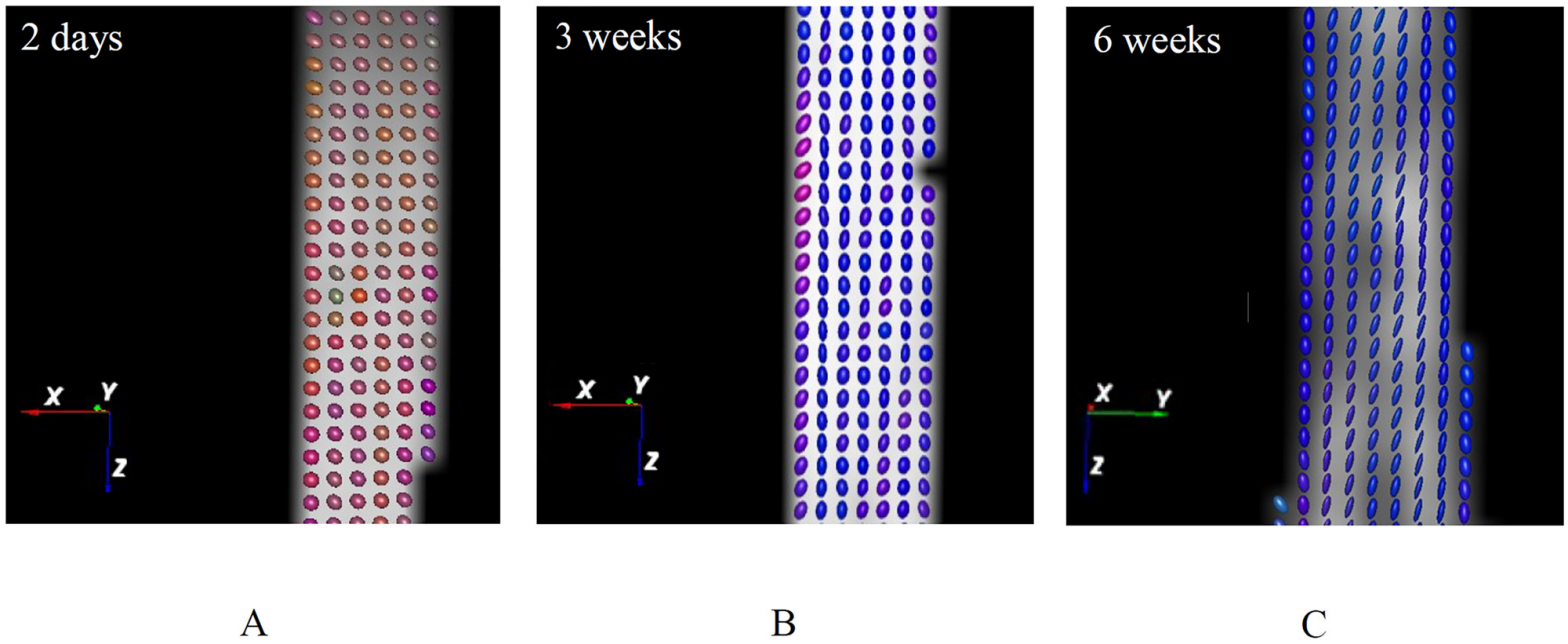
Arrays of ellipsoids obtained from the diffusion tensor of DTI scan in a single slice of middle part (10×3 mm) of TE samples after 2 days (A), 3 weeks (B) and 6 weeks of culture (C). Ellipsoids show the orientation and magnitude of the diffusion in each voxel. Fibers were randomly oriented after 2 days, but they become aligned in the constrained direction after 3 weeks and in the oblique direction in more compacted regions after 6 weeks.

**Fig 7:**
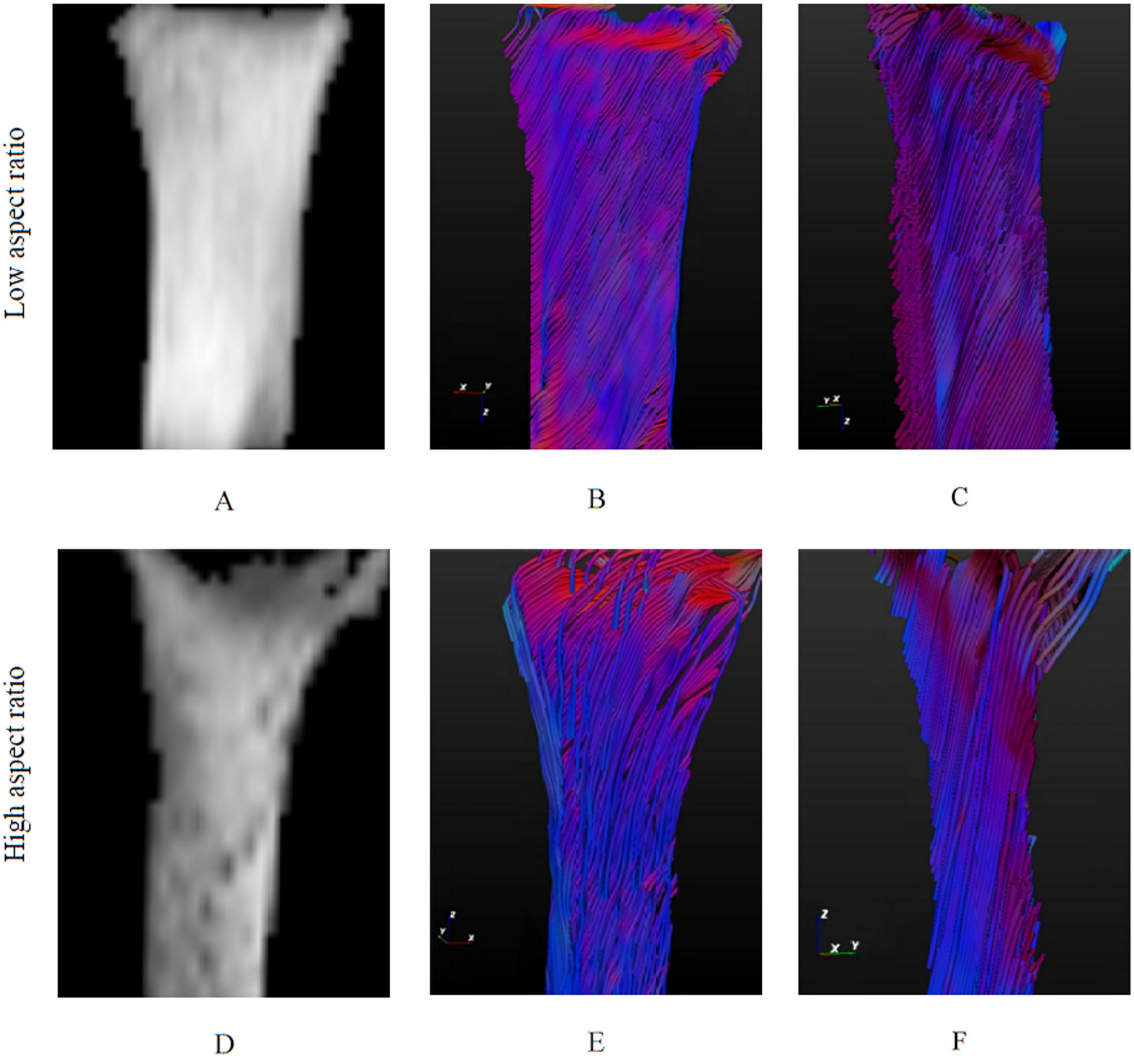
T_2_-weighted DTI images of low (A) and high (D) aspect ratio TE strips after 3 weeks of culture. Fiber tracts represent the fibrous structure in the engineered constructs with low (top view (B), side view (C)) and high (top view (D), side view (F)) aspect ratio. Fibers are color-coded based on their directions: red (x direction), green (y direction) and blue (z direction). Collagen fibers are mostly aligned in the constrained direction in high aspect ratio strips and in the oblique direction in low aspect ratio strips. Strip size in image (A-C): 10×5×1 mm, and in image (D-F): 10×3×1 mm.

The diffusion ellipsoids representing the diffusion directions are shown in Fig 6 for one slice of the high aspect ratio TE strips cultured for 2 days, 3 and 6 weeks. Ellipsoids are used to visualize the fibers directionality. The random ellipsoids orientation in the strips after 2 days of culture indicate that there was no preferred fibrous direction in the tissue at that time point (Fig 6A). Ellipsoids in the samples cultured for 3 weeks were aligned in the constrained direction (Fig 6B) and became oblique after 6 weeks of culture (Fig 6C). T_2_-weighted images and the corresponding fiber trajectories of low (1:3) and high (1:5) aspect ratio TE constructs cultured for 3 weeks are shown in Fig 7. The fibers were mainly oriented at an oblique angle to the longitudinal z-axis in the low aspect ratio samples‎ according to the fibers color, which is based on the direction of the strongest diffusion,‎ and in the constrained direction in the high aspect ratio samples. The color-coded fibers appear in the same color in Fig 7B, 7C, 7E and 7F, showing that most fibers are running roughly in the same direction.

### Quantitative comparison of CLSM and DTI data

**Fig 8:**
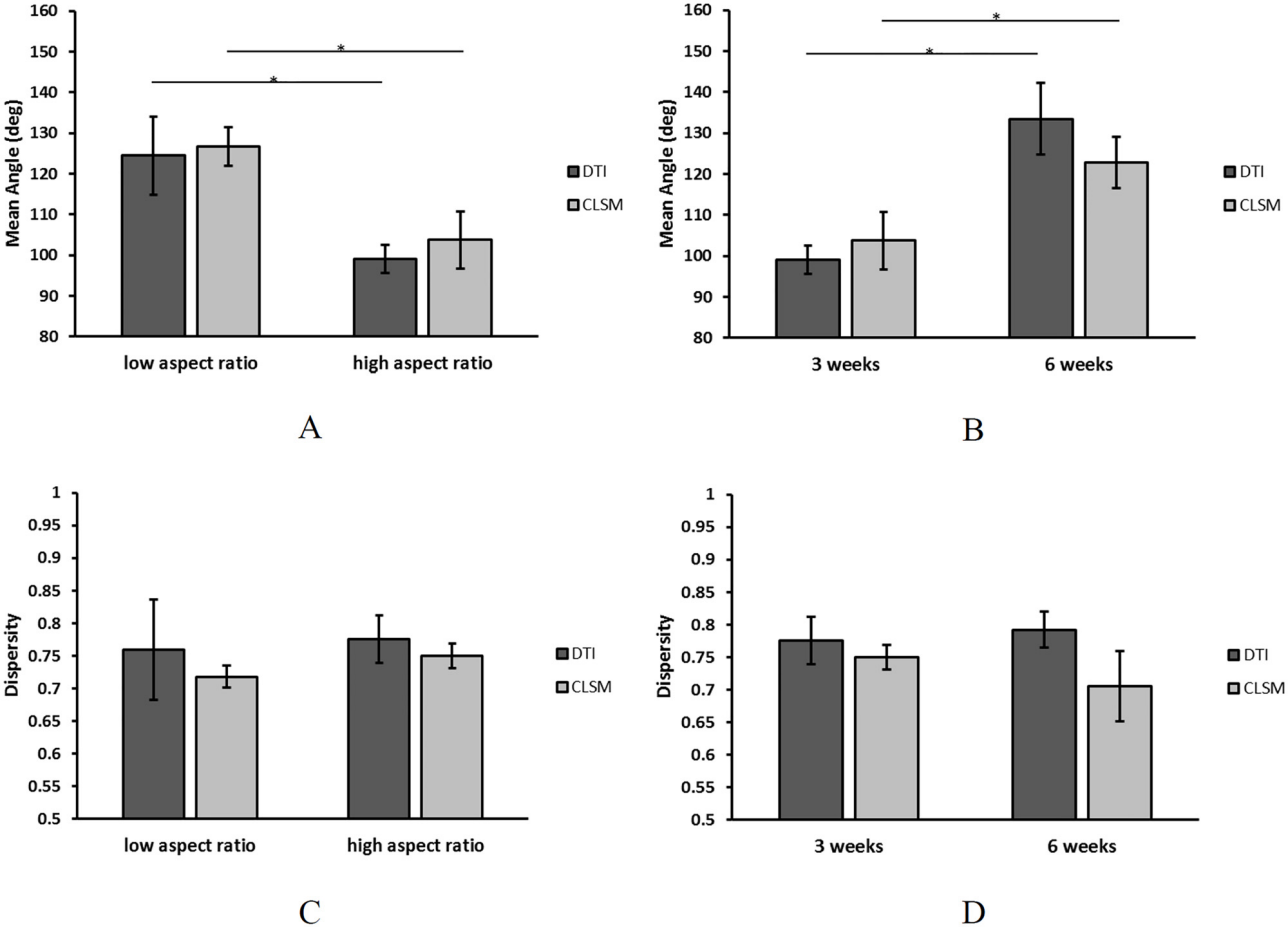
The mean angle and dispersity of fiber alignment of low aspect ratio and high aspect ratio constructs after 3 weeks of culture (A,C) and high aspect ratio after 3 and 6 weeks of culture (B,D). The mean angle of high aspect ratio group compared to low aspect ratio group and 6 weeks group compared to 3 weeks group was significantly changed. However, the dispersity was not significantly different between any of those groups. There was no significant difference between DTI and CLSM within any group. Each bar represents the mean ± SD (* represents p<0.05).

The mean angle of fiber orientation was significantly decreased in high aspect ratio strips (DTI: mean = 99.1 degrees; CLSM: mean = 103.7 degrees) compared to low aspect ratio ones (DTI: mean = 124.6 degrees; CLSM: mean = 126.7 degrees) at 3 weeks (p<0.05) (Fig 8A) and was significantly increased by extending culture time from 3 weeks (DTI: mean = 99.1 degrees; CLSM: mean = 103.7 degrees) to 6 weeks (DTI: mean = 133.7 degrees; CLSM: mean = 123.1 degrees) (p<0.05) (Fig 8B) in both methods. No preferred fiber alignment was observed at the beginning of culture time (data not shown). There were no significant differences in dispersity between the low aspect ratio (DTI: mean = 0.76; CLSM: mean = 0.72) and high aspect ratio (DTI: mean = 0.77; CLSM: mean = 0.75) at 3 weeks (Fig 8C), nor between the groups at 3 weeks (DTI: mean = 0.77; CLSM: mean = 0.75) and 6 weeks (DTI: mean = 0.79; CLSM: mean = 0.70) (Fig 8D) groups. DTI and CLSM data were not significantly different within any group.

### Histology

**Fig 9:**
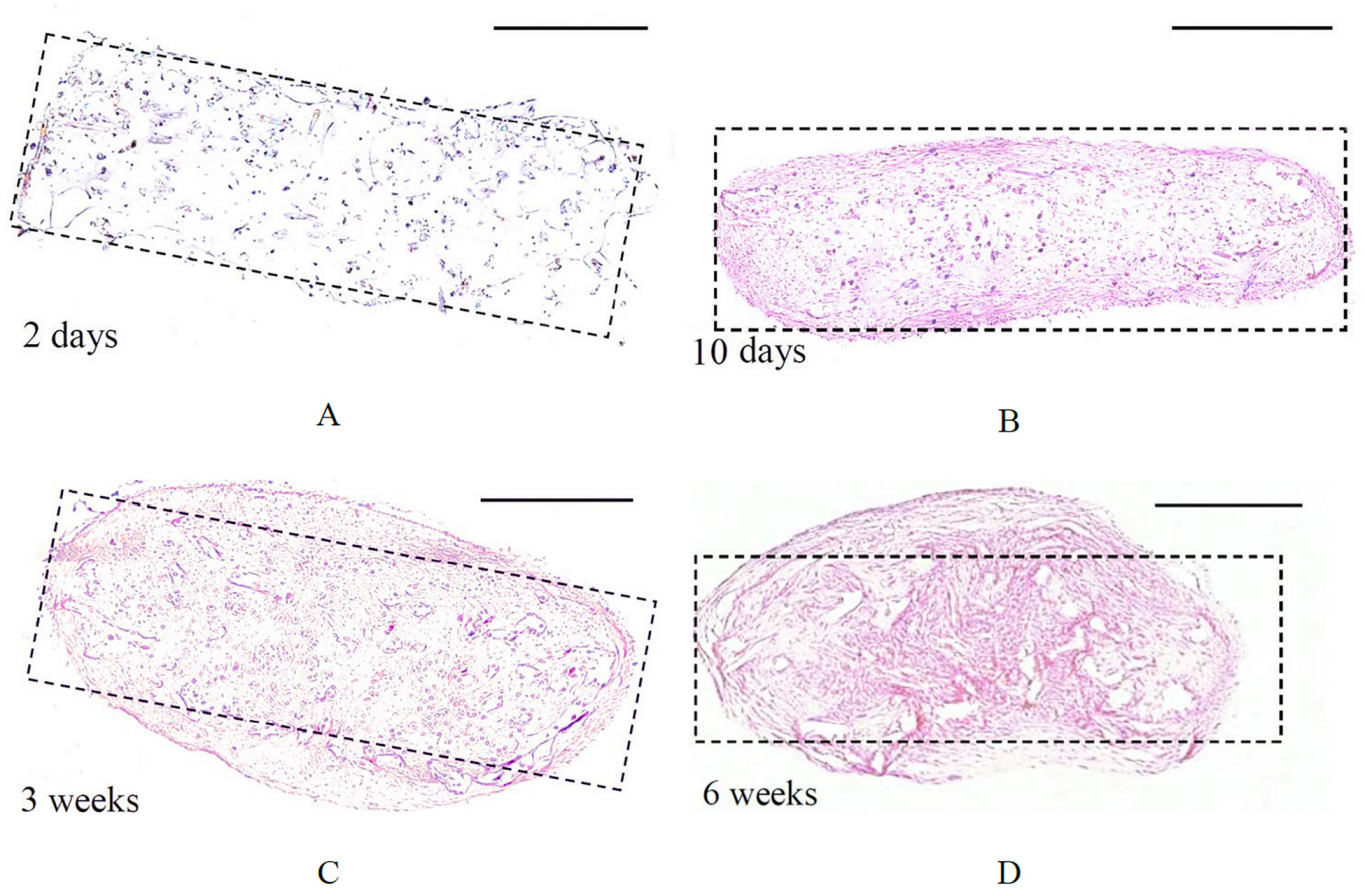
Hematoxylin and eosin staining of high aspect TE strips cultured for 2 days (A), 10 days (B), 3 weeks (C) and 6 weeks (D). The scale bar represents 500 μm. By extending culture time, TE strips became more compacted. Dashed rectangles represent the original shape of the strips.

**Fig 10:**
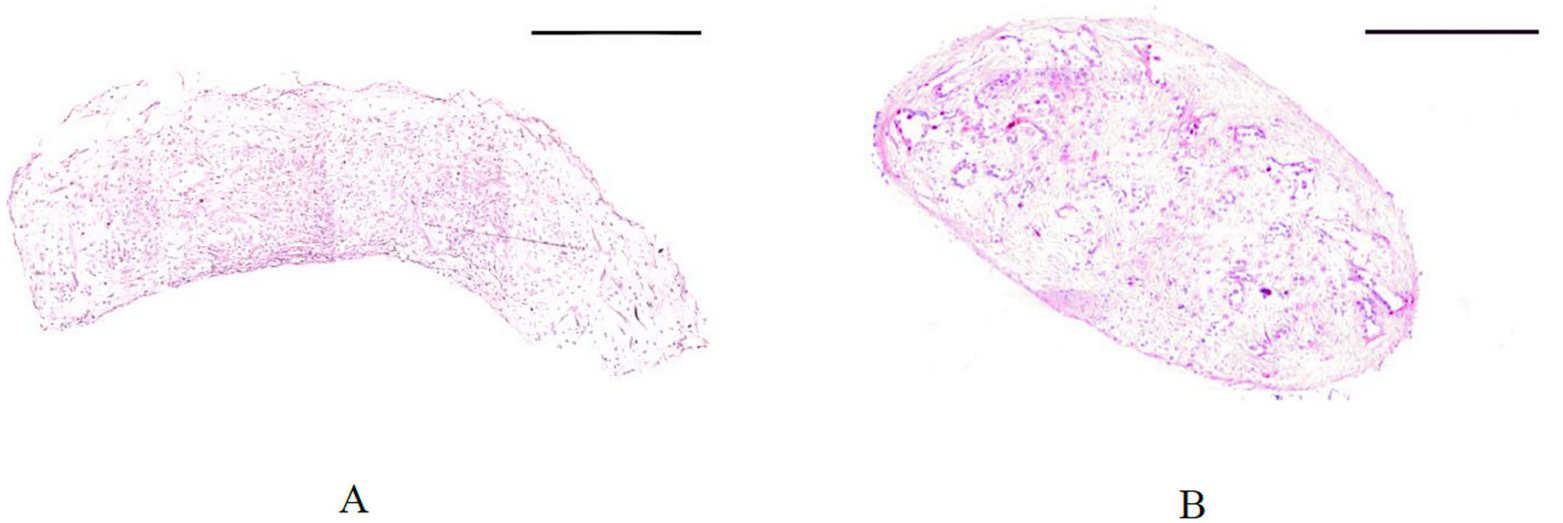
Hematoxylin and eosin staining of low (A) and high (C) aspect ratio TE sample after 3 weeks of culture. Both samples were compacted after 3 weeks. The scale bar represents 500 μm.

H&E staining of slices representing the cross-sectional area of the strips shows the rectangular shape of a typical representative example of TE strips after 2 days of culture, which became more round due to tissue compaction after extending the culture time to 6 weeks (Fig 9). This observation was in agreement with the observed reduction in width of the samples when visualized from top view using the imaging modalities. Fig 10 shows the H&E staining of high and low aspect ratio samples. Both samples demonstrated compaction after 3 weeks of culture.

## Discussion

**Fig 11:**
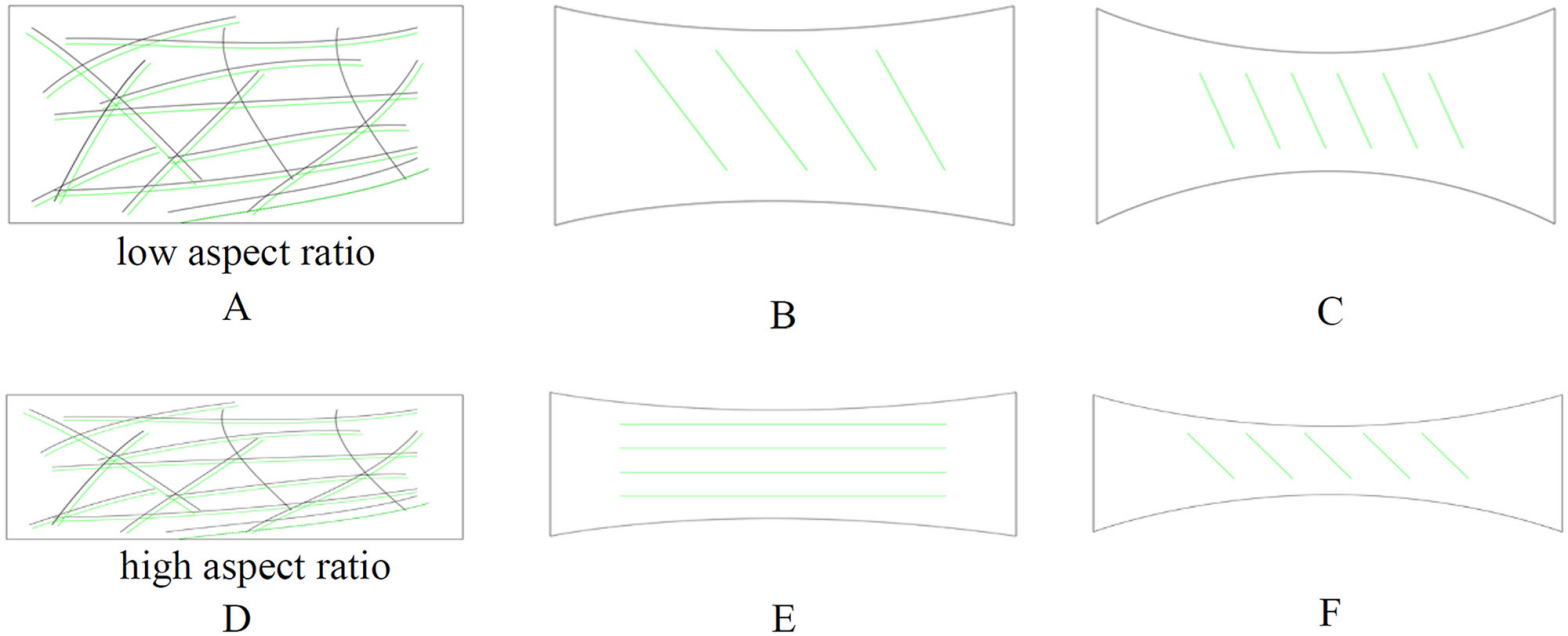
Schematic representation of collagen orientation evolution with time in low (A-C) and high (D-F) rectangular TE strips. The collagen orientation changes by culture time. First, the fibers were randomly oriented. By extending culture time, fibers aligned in the constrained direction in the high aspect ratio strips and in the oblique direction in low aspect ratio strips. By culturing for a longer time, the fibers aligned in the oblique direction in both configurations.

The collagen alignment in TE constructs plays an important role in developing the anisotropic mechanical behavior of the tissue [3]. It is crucial to understand the collagen orientation in order to make TE constructs with structures similar to the native tissue [41]. In this study, a general picture arose regarding the evolution of the collagen architecture. As depicted schematically in Fig 11, the collagen fibers were randomly distributed along the scaffold fibers in both configurations. When the scaffold degrades, the fibers aligned predominantly in the oblique direction in low aspect ratio samples and in the direction of the constraint in the high aspect ratio samples. The histology images showed that the low aspect ratio samples cultured for 3 weeks have already undergone considerable compaction in contrast to the high aspect ratio samples, which might explain the difference between the collagen orientations of high and low aspect ratio samples cultured for 3 weeks. In Figs 5 and 7, collagen fiber orientation between low and high aspect ratio samples was only compared after 3 weeks of culture as the major difference in collagen orientation between these two configurations was observed at that time point. By accentuating the compaction the fibers oriented in the oblique direction in both configurations. The collagen orientation in high aspect ratio samples was in the constrained direction and it changed toward the oblique direction by extending culture time. Both CLSM and DTI showed similar collagen alignment, confirming that DTI can be used as a fast and non-invasive method to visualize collagen orientation in TE constructs.

We directly compared CLSM and DTI as feasible imaging modalities for quantifying TE cardiovascular remodeling (Table 1). CLSM imaging provided more detailed information about the collagen orientation throughout the tissue, but had limited imaging depth. Therefore, use of CLSM to study the collagen orientation throughout the tissue would require the laborious procedure of sequential sectioning and imaging. In contrast, DTI has the potential advantage of providing faster evaluation of collagen orientation in *ex-vivo* tissue samples, albeit with a lower resolution. It can give us a good indication of the spatial (an)isotropy, i.e. in different regions of the tissue. For tissues with clear anisotropy, this method can hence be used to show the tissue 3D fibrous arrangement.

**Table 1:** Comparison of the pros and cons of CLSM and DTI modalities. Overall, DTI has more advantages to study TE cardiovascular remodeling.

Imaging technique	Pros	Cons
**3D CLSM**	Higher resolution (~ 1–2 μm)	Longer scanning time compared to DTI, Limited imaging depth (~ 200 μm), Destructive, Requirement of a proper fluorescent stain
**DTI**	Shorter scanning time compared to CLSM, No imaging depth limitation, Non-destructive, Non-invasive, No need for staining, Future *in-vivo* application	Lower resolution (~ 100 μm)

We suggest that orientation changes in the TE strips are due the ongoing tissue compaction since changes were accentuated in the most compacted region. After about 10 days, the scaffold was mostly degraded and stress generated by cells allowed for tissue compaction. The compaction forces exerted by the cells may explain why the collagen fibers realign in that direction [42]. The mechanism of collagen fiber orientation in TE constructs is not completely understood yet. Several theories have been developed to predict the collagen remodeling of cardiovascular and other soft tissues [43]. When the tissue is constrained and statically cultured, the tissue significantly compacts [11] and generated stresses and strains in the tissue are believed responsible for collagen alignment. The computational model developed by Soares et al. can predict the fiber orientation in statically cultured strips [41]. A model was developed by Driessen et al. that could successfully predict the fiber orientation of native arterial wall and heart valve, but not TE constructs [44]. The model was extended by adding volumetric growth and hypothesized that collagen fibers orient based on the stresses and strains, which were generated during tissue development and compaction [41]. They showed that the stress in the longitudinal direction and the compaction width of the statically cultured strips increased over time. This model could also predict that the collagen fibers align in the direction of the constraint, which coincides with the maximum principal stress direction, but was unable to predict the observed oblique orientation.

In conclusion, collagen orientation evolution with time in engineered tissues of both low and high aspect ratio geometries was successfully evaluated by DTI. CLSM, as an accepted method for collagen fiber visualization, validated the DTI results. Thus, DTI can be used to quickly evaluate collagen orientation evolution in 3D engineered tissues. The validity of this method for *in-vivo* applications should be checked in future pre-clinical studies. This would help us to evaluate and understand collagen remodeling in the in‐vivo environment and under physiological conditions.
